# Mr. Coulson on Paralysis of the Bladder

**Published:** 1842-12-17

**Authors:** 


*Mr. Coulson on Paralysis of the Bladder.*—This complaint often attacks elderly persons, particularly those who are gouty and rheumatic. It is the result of general diminution of nervous and muscular power; the bladder being less sensible to the stimulus of the urine, and being incapable of obeying the will with the same facility as before.

Paralysis of the bladder is peculiarly, as Soemmering observes, a disease of old age; the excitability of the organic formation, particularly that of the muscular fibres, gradually decreases; the susceptibility of the nerves becomes limited, and the cellular membrane loses its tone. Hence, an attack of paralysis is often long announced by weakness in the loins, tottering in the step, and bending of the knees in walking. From this it may almost always be inferred, that a considerable alteration in the functions of the spinal marrow, and of the nerves proceeding from it and from the sacrum to the bladder, has taken place.

The bladder is rendered incapable of obeying the will with the same facility as before: no longer so susceptible of the customary excitement of the urine, it yields to the urine, and becomes distended, sometimes to an enormous size, even so much as to burst, when the patient almost immediately expires. Haller saw a bladder in a great drinker, which was able to contain twenty pounds' weight of water. Frank found a bladder so distended as to give the appearance of dropsy ; twelve pounds of urine passed at one time through the catheter, although he did not remove the whole of it.

Baillie remarks that this expansion of the bladder may arise from an accidental circumstance, where the urine accumulates whilst the muscular coat still retains its peculiar power; or the muscular coat of the bladder may likewise be paralysed, and hence be incapable of expelling the urine. The difference between these two circumstances can always be ascertained by an examination of the case during life.

Children are likewise subject to both partial and complete paralysis of the bladder. In some cases, the urine continues to pass involuntarily by day as well as by night ; and then the patient suffers more or less from this complaint during the remainder of his life. When this occurs only in sleep, children should be waked up in the night, twice or oftener, for the purpose of passing urine ; and they should not be permitted to take late meals, or much liquid for some time prior to retiring to rest. Indeed, they should not at any time be allowed to take much liquid. As lying on the back in bed tends to keep up this complaint, it should be guarded against. Cold bathing, preparations of iron, and other tonics, are of great use. This complaint usually improves as children get older, and often disappears after puberty, owing to the increased energy which the genito-urinary organs then experience. If it continue after puberty, the tincture of cantharides is often very serviceable, together with the occasional application of blisters across the sacrum or loins.

M. Lallemand strongly recommends the use of baths, medicated with aromatic plants ; and mentions several cases which were cured by using them. After eight or ten baths the beneficial effects are usually observed, and fifteen or eighteen generally suffice for the cure. Sometimes the complaint returns after a few months ; if so, the baths must be again resorted to.

I have known this state to occur after an attack of scarlet fever. A boy, now seventeen years of age, was quite well till ten years ago, when he was attacked with scarletina ; and ever since that period he has been afflicted with incontinence of urine during the night.

Incontinence of urine is frequently connected with a weak and scrofulous state of constitution ; and often, in these cases, all remedies are unavailing. I saw some time ago a child, six or seven years old, with a large head, pale countenance, bad teeth, prominent sternum, large abdomen, and emaciated extremities, who, from its earliest infancy, had suffered from incontinence. All kinds of remedies had been tried without success. In this case, as in many others, there was great irritability of the bladder during the day, and, unless the desire to pass urine was attended to, it flowed off involuntarily.— The urine was very acid.

In incontinence of urine, it is of the utmost importance to preserve the patient from the consequences of a perpetual dribbling of the urine. For this purpose, a receptacle should be worn during the day to contain the urine as it is passed. The contrivances in ordinary use, composed of gum elastic, are objectionable, on account of the strong urinous smell which they soon acquire, notwithstanding every attention to cleanliness. An oval glass bottle or vessel composed of copper or platina, and covered with a piece of thin macintosh, is better adapted for persons laboring under this complaint. These contrivances are made for females as well as males. During the night, patients should have a piece of macintosh cloth placed under them.

In some persons, particularly in stout females, the urine will flow involuntarily on lifting a weight, or coughing, or any violent exercise. In such cases, there is no pain, no blood in the urine, no desire to make water often, but merely an involuntary flow on exertion; the action of the diaphragm and abdominal muscles overcoming that of the sphincter of the bladder. In females, incontinence often occurs after distension of the urethra for the extraction of calculi or foreign bodies, and occasionally after difficult labours. Dr. Cory relates a rather interesting case of the latter, which speedily yielded to one-sixteenth part of a grain of strychnia, given three times a day. In these cases, it is probable that the sphincter vesicæ has been injured.

In the end of gestation, incontinence of urine is not uncommon, being produced by the pressure of the uterus on the bladder, by which the urine is expelled involuntarily, whenever the woman coughs or moves quickly; at least she cannot retain much of it, being obliged to void it frequently, though without stranguary. For this complaint there is no cure; and many regard it favorably, as an omen that the child's head is resting on the os uteri. When the uterus is very pendulous, some advantage may, in this respect, be obtained, by supporting the belly with a proper bandage, attached to the shoulders.

Incontinence of urine, resulting from a mere weakness of the neck of the bladder, is common in those who have had very large families, ten or twelve children, for example. In these cases, more especially if the child is large, or the pelvis small, when the labour has been severe, the bladder is apt to get so infirm about the neck, that it loses much of its retractive power, and, perhaps, from the moment of delivery, the woman is incapable of retaining the water: or if, at any time she chance to cough, laugh, rise suddenly, or in any other manner contract smartly the abdominal muscles, the water comes gushing away. For years this disease may continue with greater or less severity, but it frequently cures itself, in good measure, and the first few weeks after delivery, say at the end of the fortnight, the patient is better; at the end of the month the retentive powers are still more increased; and in the course of a few more weeks she becomes able to hold the water very well, though still liable to gushes, when sudden efforts are made. Hence, where inconvenience is the result of an enfeebled cervix vesicæ, time must be looked upon as one of the principal remedial means; in some cases, advantage may be obtained from plunging the hips into cold water two or three times daily. The improvement of the general health is by no means to be neglected, for the more you improve the general health, the more you will increase those healing powers of the parts on which all cures are more immediately dependent.—*On Diseases of the Bladder, and Prostate Gland, &c.*, pp. 59–64.

